# Health-Specific Information and Communication Technology Use and Its Relationship to Obesity in High-Poverty, Urban Communities: Analysis of a Population-Based Biosocial Survey

**DOI:** 10.2196/jmir.5741

**Published:** 2016-06-28

**Authors:** Anjali Gopalan, Jennifer A Makelarski, Lori B Garibay, Veronica Escamilla, Raina M Merchant, Marcus B Wolfe Sr, Rebecca Holbrook, Stacy Tessler Lindau

**Affiliations:** ^1^ Corporal Michael J. Crescenz VA Medical Center Philadelphia, PA United States; ^2^ Kaiser Permanente Northern California Division of Research Oakland, CA United States; ^3^ Robert Wood Johnson Clinical Scholars Program Perelman School of Medicine at the University of Pennsylvania Philadelphia, PA United States; ^4^ The University of Chicago Department of Obstetrics and Gynecology Chicago, IL United States; ^5^ University of Chicago Chicago, IL United States; ^6^ Concordia University-Chicago Chicago, IL United States; ^7^ Holbrook Consulting Chicago, IL United States; ^8^ The University of Chicago Department of Obstetrics and Gynecology, and Medicine-Geriatrics Chicago, IL United States; ^9^ The University of Chicago Comprehensive Cancer Center Chicago, IL United States; ^10^ The University of Chicago Maclean Center on Clinical Medical Ethics Chicago, IL United States; ^11^ The University of Chicago Urban Health Initiative Chicago, IL United States

**Keywords:** obesity, technology, Internet, urban health

## Abstract

**Background:**

More than 35% of American adults are obese. For African American and Hispanic adults, as well as individuals residing in poorer or more racially segregated urban neighborhoods, the likelihood of obesity is even higher. Information and communication technologies (ICTs) may substitute for or complement community-based resources for weight management. However, little is currently known about health-specific ICT use among urban-dwelling people with obesity.

**Objective:**

We describe health-specific ICT use and its relationship to measured obesity among adults in high-poverty urban communities.

**Methods:**

Using data collected between November 2012 and July 2013 from a population-based probability sample of urban-dwelling African American and Hispanic adults residing on the South Side of Chicago, we described patterns of ICT use in relation to measured obesity defined by a body mass index (BMI) of ≥30 kg/m^2^. Among those with BMI≥30 kg/m^2^, we also assessed the association between health-specific ICT use and diagnosed versus undiagnosed obesity as well as differences in health-specific ICT use by self-reported comorbidities, including diabetes and hypertension.

**Results:**

The survey response rate was 44.6% (267 completed surveys/598.4 eligible or likely eligible individuals); 53.2% were African American and 34.6% were Hispanic. More than 35% of the population reported an annual income of less than US $25,000. The population prevalence of measured obesity was 50.2%. People with measured obesity (BMI≥30 kg/m^2^) were more likely to report both general (81.5% vs 67.0%, *P*=.04) and health-specific (61.1% vs 41.2%, *P*=.01) ICT use. In contrast, among those with measured obesity, being told of this diagnosis by a physician was not associated with increased health-specific ICT use. People with measured obesity alone had higher rates of health-specific use than those with comorbid hypertension and/or diabetes diagnoses (77.1% vs 60.7% vs 47.4%, *P*=.04).

**Conclusions:**

In conclusion, ICT-based health resources may be particularly useful for people in high-poverty urban communities with isolated measured obesity, a population that is at high risk for poor health outcomes.

## Introduction

Obesity and obesity-related chronic diseases are leading drivers of health care costs in the United States [1]. Over the past 35 years, the prevalence of obesity has more than doubled; currently, 35% of American adults are obese (defined as body mass index, BMI, ≥30 kg/m^2^) [1]. Certain populations are disproportionately affected by obesity, including African American and Hispanic adults and people living in resource-poor, high-poverty, and more racially segregated urban communities [1-5]. Many major technology corporations, payers, and health care systems are investing in information and communication technologies (ICTs), such as mobile apps and Web-based patient portals, to prevent and better manage obesity and related chronic conditions [6-10]. Growing, but limited, evidence demonstrates that ICT-based interventions can positively affect health behaviors and obesity-related outcomes [6,11,12]. Small, clinic-based trials of mobile apps and other Web-based decision support and monitoring tools have demonstrated improved outcomes for specific chronic health conditions, including short-term weight loss [6,11]. Although these findings are promising, the ability of health-specific ICT-based resources to impact health outcomes will depend not only on efficacy in clinical trials but on actual use among people with obesity, especially those residing in communities with limited health resources [13].

The 2012 Pew Internet Health Tracking Survey examined the relationship between types of ICT use, including seeking information online about conditions, medications, or the experiences of others, and self-reported chronic disease [14]. In this study, controlling for age, income, education, ethnicity, and overall health status, people who self-reported a diagnosis of chronic disease, including hypertension, diabetes, heart, and lung disease, were less likely to report any ICT use (the frequency of these activities was not described) [14]. However, among people who did report ICT use, those with one or more chronic diseases were more likely to report the use of ICT for health-specific reasons compared with those without a chronic condition [14]. Obesity, designated by the American Medical Association as a chronic condition after the study’s completion, was not included among the chronic conditions examined in the Pew survey [15]. Also missing from the Pew survey are any biometric data regarding chronic disease status, specifically BMI. A population-based survey that collected individual-level data on general and health-specific ICT use and chronic disease status, including both self-reported obesity diagnoses and objective obesity status (anthropometric measures) presented an opportunity to address these gaps in the Pew data [16].

On Chicago’s South Side, 55% of the population (approximately 528,000) lives at or below 200% federal poverty level; 77% of residents are African American, 13% are Hispanic [17]. African American and Hispanic people have disproportionately high rates of obesity [2]. Vital statistics data for the region suggest higher rates of premature and overall mortality related to obesity-related chronic diseases compared with more affluent areas of Chicago [18]. As part of a larger strategy to mitigate health inequities in the region, community leaders, residents, and university researchers with the South Side Health and Vitality Studies conducted a population health survey to establish prevalence estimates for obesity and other chronic diseases and to ascertain what kinds of community-based and ICT resources residents use to manage their health [19]. We hypothesized that residents use ICT resources to substitute for gaps in community-based resources and that ICT use varies by health condition. Understanding the feasibility of ICT-based health resources to reach people with obesity in high-poverty communities is critical to maximizing the potential of these resources to impact health.

In this study, we first describe overall patterns of current ICT use by measured obesity status. Based on the Pew findings for other chronic conditions, we hypothesized that although overall ICT use would be lower among respondents with measured obesity, health-specific ICT use would be higher. Second, among people with measured obesity (BMI≥30 kg/m^2^), we described health-specific ICT use comparing those with and without a physician’s diagnosis of obesity and by the presence of self-reported comorbid conditions, specifically diabetes and hypertension. The Health Belief Model suggests that willingness to perform a health behavior, such as using health-specific ICT resources, depends on the perceived need for action [20]. Thus, obese people who were never told by a physician they had obesity or an obesity-related comorbid condition may be less motivated to use ICT-based health resources than people with a physician’s diagnosis or who have one or more of these comorbid diagnoses [20]. We, therefore, hypothesized that health-specific ICT use would be higher among people with BMI≥30 kg/m^2^ who have been diagnosed as obese by a physician and those with measured obesity who also self-report a diagnosis of comorbid hypertension and/or diabetes. Lastly, based on the study findings and extant literature, we propose a conceptual framework describing the relationship between obesity and health-specific ICT use that we hope will inform the design and translation of ICT-based interventions targeting obesity management.

## Methods

This analysis is based on data from the South Side Health and Vitality Studies (SSHVS) [16]. SSHVS is a family of interrelated, community-engaged research studies that aim to inform efforts to promote and maintain population health on Chicago’s South Side [21]. SSHVS aims to describe population health in the region and the ways in which residents use ICT to access health-related community resources or substitute for gaps in local resources. All participants provided written documentation of informed consent. This study was designed in partnership with volunteer community members [19] and was approved by the University of Chicago Institutional Review Board (IRB). The primary data collection for this research and the activity of the University of Chicago researchers were conducted under the approved University of Chicago IRB protocol. Secondary data analysis was conducted by AG following a human subjects research exemption granted by University of Pennsylvania IRB. Other co-authors had no access to the study subjects or individual-level data.

### Study Population

Individuals eligible for this study included those 35 years of age or older, English or Spanish speaking, and residing within the target region.

### Sampling

Study participants were sampled from 2 distinct regions, a total of 7 census tracts, on the South Side of Chicago. The northwest region was almost entirely African American (98%), based on 2010 US Census data [22]. The southeast region was majority Hispanic (83%). We employed a single-frame, two-stage sampling design. First, an address-based probability sample of household units was generated by randomly selecting household units from a list of all residential postal addresses in the regions purchased from Marketing Systems Group’s Genesys Division, 2012 [23]. Then, if more than one individual in a household was 35 years or older, one individual was randomly selected.

### Data Collection

Eligible participants were recruited between November 2012 and July 2013 through mailed letters, telephone calls, and home visits. Once informed consent was obtained, participants took part in an in-person, interviewer-administered structured interview, lasting approximately 1 hour. Participants could choose to complete the interview in English or Spanish. The interview collected sociodemographic characteristics, details on ICT device ownership and use, and self-reported medical history. Physical measures were obtained, including height, weight, waist circumference, systolic and diastolic blood pressure, and finger-stick dried blood specimens.

### Defining Information and Communication Technology Use

General ICT use was defined as any use of cell phones for texting, emailing, going online, or downloading apps, or any use of the Internet (accessed via computer or cell phone). Health-specific ICT activities included the following activities: looking up health information online, using a health-related mobile app, Web-based purchasing of medications, Web-based communication with providers, participation in online health support groups, and Web-based management of health records and/or benefits. Questions included on the survey instrument relating to health-specific ICT use were primarily based on a prior national-level survey, the Health Information National Trends Survey, which was modified by the study team to increase cultural appropriateness and aid comprehension (Textbox 1) [24]. On the basis of review of our survey instrument by community informants, an additional question was added asking those who reported looking up health information online the following question: “Do you ever use the Internet to find health information because you did not want to ask a doctor?” This question was included to explore a hypothesis that people might use the Internet for information to avoid embarrassing discussions or to compensate for limited time with health care providers. For all of these health-specific activities, use was primarily defined as any amount of engagement (ranging from daily to less than monthly) or no engagement. The distribution of activity frequency was also described. The dichotomous categorization of health-specific ICT use was done to allow examination of associations between selected activities and participant characteristics (small cell counts would prevent statistical testing) and because the “right” amount of each of the examined activities is unknown and likely varies greatly. This categorization also reflected that used in the aforementioned Pew survey that studied ICT use and self-reported chronic conditions [14].

**Survey instrument used to assess selected health-specific information and communication technology activities (questions 1-5 were modified from the 2003 Health Information National Trends Survey, item HC-26, and question 5a was constructed based on input from community stakeholders).**

Instructions: I’m going to list some ways people use the Internet. Some people have done these things and some have not. Please use Card #X to tell me how often you did these things in the past 12 months. In the past 12 months, how often have you...

□ Every day □ At least once a week □ At least once a month □ <Once a month □ Never

1. Bought some kind of medicine online. This includes prescription medicines, over-the-counter medicines, or herbal supplements?

2. Taken part in an online support group for people with a health or medical issue?

3. Used e-mail or the Internet to talk with a doctor or a doctor’s office or hospital?

4. Looked for health or medical information online?

5. Looked at or managed your health records online

5a: If yes, do you ever use the Internet to find health information because you did not want to ask a doctor?

□ Yes □ No □ Don’t Know □ Refused

6. Looked at or managed your health benefits, like filing an insurance claim online

### Defining Obesity and Other Chronic Disease Variables

**Figure 1:**
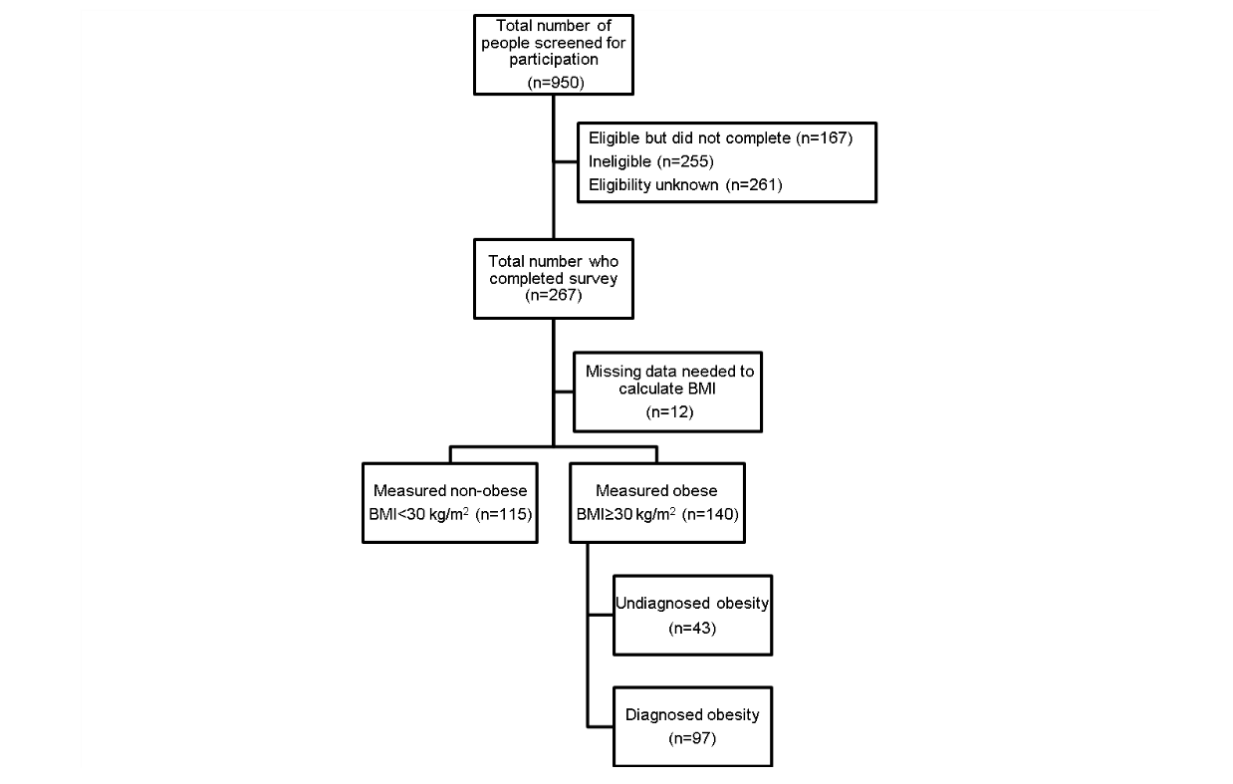
Population-based probability sample enrollment flowchart. BMI: body mass index.

Body mass index was calculated using measured height and weight collected during in-home interviews [25]. For the primary analysis, measured obesity was defined as a binary variable: nonobese (BMI<30 kg/m^2^) and obese (BMI≥30 kg/m^2^; Figure 1). Those with missing BMI data (n=12) were excluded. To examine the effect of being diagnosed as obese by a physician, respondents who were categorized as obese by their measured BMI were then stratified by their response to the survey question “Has a medical doctor ever told you that you have excess weight or obesity?” (Figure 1). The resultant binary variable categories were labeled “diagnosed obese” and “undiagnosed obese.” To assess differences based on a diagnosis of comorbid chronic conditions, diabetes and hypertension were defined as binary variables based on survey responses to the following two questions: (1) “Has a medical doctor ever told you that you have diabetes?” and (2) “Has a medical doctor ever told you that you have high blood pressure or hypertension?” Given the significant overlap between the 3 examined conditions, the 3 categories were defined as follows: measured obesity only, measured obesity plus self-reported hypertension or diabetes, and measured obesity, self-reported hypertension, and self-reported diabetes.

### Statistical Analysis

All analyses for this population-based probability sample were weighted to account for differential selection probabilities and differential nonresponse. The response rate and the cooperation rate were calculated using the American Association for Public Opinion Research (AAPOR) definitions for response rate (RR3) and cooperation rate (COOP3) [26]. The response rate describes the number of completed surveys in relation to the total number of eligible individuals (we assumed an eligibility rate of 63% for 261 individuals of unknown eligibility; Figure 1), whereas the cooperation rate describes the number of completed surveys in relation to the number of eligible individuals ever contacted [26]. Descriptive statistics were calculated to summarize sociodemographic characteristics by obesity status and diagnosis of obesity. Differences in general and health-specific ICT use among the people with and without measured obesity were examined. Among those with measured obesity, the associations between health-specific ICT use and diagnosis of obesity were also compared. Lastly, differences in health-specific ICT use among those with measured obesity only were compared with those reporting a diagnosis of hypertension or diabetes and those with all 3 conditions. Chi-square tests were used to test for statistical differences between or among groups. All data analyses were performed using Stata version 12.1 (2011, StataCorp, College Station, TX, USA).

## Results

The response rate was 44.6% (267 completed surveys/598.4 eligible or likely eligible individuals) and the cooperation rate was 61.5% (267 completed surveys/434 eligible individuals contacted). In total, 267 individuals participated in the biosocial study.

### Sociodemographic Characteristics and ICT Use by Measured Obesity Status

Associations between sociodemographic characteristics and measured obesity (BMI≥30 kg/m^2^) status in the population are summarized in Table 1. Measured obesity was more prevalent among women than men (63.2% vs 36.8%, *P*=.01). Measured obesity status did not differ by income, education, or race or ethnicity. The majority of people reported seeing a physician in the past year (79.8%) and this did not differ by measured obesity status (82.5% obese vs 78.6% nonobese, *P*=.55). However, individuals with measured obesity were more likely to report a source of regular care than those without obesity (96.7% obese vs 88.6% nonobese, *P*=.04).

The prevalence of general ICT use in the population was high (75.0%) but was more common among people with obesity when compared with people without obesity (81.5% obese vs 67% nonobese, *P*=.04; Table 2). Half of all respondents (51.7%) reported some type of health-specific ICT use, but it was more common among people with measured obesity (61.1% obese vs 41.2% nonobese, *P*=.01). The most common health-specific ICT activity in the population was looking up health-related information online (47.2%); this activity was more common among the people with obesity (54.4% obese vs 38.7% nonobese, *P*=.04). Other health-related activities, including Web-based medical record access (8.8%), participation in online health-related support groups (9.3%), and Web-based communication with providers (9.7%), were infrequent and did not differ by obesity status (Table 2). Health-specific mobile app use was also uncommon (7.6%) and did not differ by measured obesity status. A supplementary table describes the distribution of frequencies for the examined health-specific ICT activities (see Multimedia Appendix 1).

### Sociodemographic Characteristics and ICT Use by Obesity Diagnosis

Among people with measured obesity, a physician’s diagnosis of obesity was associated with educational attainment; people with obesity who had been diagnosed by a physician were more likely to have achieved a high school diploma or have passed a general educational development (GED) test (41.2% diagnosed vs 24.6% undiagnosed, *P*=.01) or some college experience (41.2% diagnosed vs 26.0% undiagnosed, *P*=.01; Table 3). Obese individuals with an obesity diagnosis were also more likely to report a source of regular health care (100% diagnosed vs 89.1% undiagnosed, *P*=.01). Obesity diagnosis was similar across the other examined sociodemographic characteristics (Table 3). Rates of ICT use by obesity diagnosis were not significantly different for general (85.6% diagnosed vs 72.0% undiagnosed, *P*=.15) or health-specific use (65.9% diagnosed vs 49.8% undiagnosed, *P*=.15). Although no statistically significant differences were noted in use of Web-based resources to look up health-related information (60.7% diagnosed vs 39.6% undiagnosed, *P*=.06), people with diagnosed obesity were more likely to look up information online as a means to avoid asking a doctor (32.0% diagnosed vs 5.0% undiagnosed, *P*<.001). No other differences in rates of specific health-related ICT activities by obesity diagnosis status were noted.

**Table 1:** Sociodemographic characteristics of the population and by measured obesity status (results of weighted analysis).

Characteristic		Total population % (95% CI)	Nonobese (BMI^a^<30 kg/m^2^) % (95% CI)	Measured obese (BMI≥30 kg/m^2^) % (95% CI)	*P* ^b^
		N=267^c^	n=115 44.9 (37.7-52.2)	n=140 50.2 (43.0-57.5)	
Age, years					
	35-40	11.8 (7.5-16.0)	9.5 (3.9-15.1)	13.9 (7.3-20.6)	.42
	41-50	36.1 (28.8-43.5)	38.1 (26.8-49.3)	35.7 (25.4-45.9)	
	51-60	22.4 (17.3-27.6)	20.8 (13.3-28.3)	23.5 (16.1-30.8)	
	61-70	13 (8.2-17.7)	17.5 (9.4-25.6)	9.1 (3.3-14.9)	
	71+	16.7 (11.1-22.4)	14.1 (6.4-21.8)	17.8 (9.4-26.2)	
Gender					
	Male	42.2 (34.9-49.4)	49.5 (38.5-60.5)	36.8 (26.6-47.0)	.01
	Female	57.8 (50.6-65.1)	50.5 (39.5-61.5)	63.2 (53.0-73.4)	
Race/ethnicity					
	Black, non-Hispanic	53.2 (48.9-57.5)	51.4 (42.4-60.4)	55.2 (46.4-63.9)	.30
	Hispanic	34.6 (28.7-40.5)	39.1 (29.0-49.3)	29.4 (19.4-39.4)	
	Other	12.2 (7.8-16.6)	9.5 (3.6-15.3)	15.4 (8.4-22.4)	
Income, US $					
	<$25K	36.2 (29.5-43.0)	40.7 (30.1-51.3)	34.2 (24.8-43.5)	.59
	$25K-$49K	28.4 (22.0-34.7)	24.1 (15.4-32.8)	33.9 (24.4-43.5)	
	$50K-$99K	16.7 (11.2-22.2)	14.2 (6.7-21.8)	17.1 (8.9-25.3)	
	≥$100K	6.7 (2.8-10.7)	8.4 (1.3-15.6)	5.8 (1.2-10.4)	
	Don't know/refused	12.0 (6.6-17.5)	12.5 (3.7-21.4)	9.0 (2.4-15.5)	
Education					
	Middle school/ some high school	30.4 (23.5-37.4)	33.3 (22.6-44.0)	27.2 (17.8-36.6)	.55
	High school graduate/GED^d^	34.8 (27.8-41.8)	37 (26.7-47.4)	36.2 (26.1-46.3)	
	Associates/ some college	34.7 (28.1-41.3)	29.7 (19.9-39.5)	36.6 (27.4-45.9)	
Employment status					
	Unemployed	14.3 (9.5-19.2)	15.5 (7.7-23.2)	14.7 (8.0-21.4)	.85
	Employed	45.5 (38.2-52.7)	46.4 (35.5-57.3)	42.5 (32.3-52.6)	
	Retired	18.7 (13.2-24.1)	18.4 (10.6-26.2)	18.6 (10.6-26.6)	
	Unable to work	10.0 (5.4-14.5)	7.1 (0.4-13.8)	12.5 (5.9-19.1)	
	Other	11.6 (6.5-16.6)	12.6 (4.3-20.8)	11.8 (4.9-18.7)	
Health insurance					
	Uninsured	24.6 (18.1-31.0)	33.8 (23.0-44.5)	17.1 (9.7-24.5)	.01
	Medicaid only	8.9 (5.0-12.8)	4.5 (0.8-8.1)	13.7 (6.9-20.6)	
	Medicare only	12.4 (8.0-16.8)	15.4 (7.8-23.1)	10.8 (5.7-16.0)	
	Private/other	38.0 (31.0-44.9)	35.4 (25.0-45.7)	40.5 (30.5-50.5)	
	Multiple	16.2 (10.9-21.4)	11.0 (4.8-17.2)	17.9 (9.8-25.9)	
Physician visit in past year (% yes)		79.8 (73.6-86.0)	78.6 (69.5-87.7)	82.5 (73.6-91.4)	.55
Source of regular care (% yes)		91.4 (87.4-95.4)	88.6 (82.2-94.9)	96.7 (93.1-1.0)	.04

^a^ BMI: body mass index.

^b^*P* value for comparison between nonobese and obese populations; significant at level *P*<0.05.

^c^ A total of 12 people were missing a BMI value. These individuals were excluded from the chi-square analysis.

^d^ GED: general educational development.

**Table 2:** Information and communication technology–based activities of the population and by measured obesity status (results of weighted analysis).

ICT^a^ activities (% reported yes)		Total population % (95% CI)	Measured nonobese % (95% CI)	Measured obese % (95% CI)	*P* ^b^
		N=267^c^	n=115 44.9 (37.7-52.2)	n=140 50.2 (43.0-57.5)	
General ICT use		75.0 (68.5-81.3)	67.0 (56.7-77.3)	81.5 (73.1-90.0)	.04
Any health-specific use		51.7 (44.5-59.0)	41.2 (30.6-51.9)	61.1 (51.0-71.1)	.01
	Seek health info online	47.2 (40.0-54.5)	38.7 (28.1-49.3)	54.4 (44.1-64.6)	.04
	Use Web-based resources to avoid asking doctor	20.2 (14.8-25.7)	16.0 (8.9-23.5)	23.9 (15.7-32.1)	.17
	Web-based access of health benefit info	12.2 (7.8-16.6)	5.8 (0.9-10.7)	18.6 (11.2-26.1)	.01
	Participate in online health support group	9.3 (5.1-13.4)	8.4 (2.8-14.0)	10.5 (4.0-17.0)	.63
	Web-based access of health records	8.8 (4.9-12.7)	4.9 (−0.1 to 9.9)	12.7 (6.5-18.9)	.08
	Web-based medication purchasing	7.7 (4.3-11.1)	5.1 (0.9-9.3)	10.0 (4.5-15.5)	.17
	Web-based communication with providers	9.7 (5.9-13.5)	7.3 (2.5-12.1)	12.4 (6.2-18.6)	.20
	Use health-related mobile app	7.6 (3.8-11.4)	5.0 (−0.03 to 10.0)	9.0 (3.6-14.3)	.31

^a^ ICT: information and communication technology.

^b^*P* value for comparison between measured nonobese and obese populations; significant at level *P*<0.05.

^c^ A total of 12 people were missing a body mass index value. These individuals were excluded from the chi-square analysis.

**Table 3:** Sociodemographic characteristics of the measured obese population and by obesity diagnosis status (results of weighted analysis).

Characteristic		Total measured obese^a^ (BMI^b^≥30 kg/m^2^) % (95% CI)	Undiagnosed obese % (95% CI)	Diagnosed obese % (95% CI)	*P* ^c^
		n=140 50.2 (43.0-57.5)	n=43 30.1 (20.7-39.5)	n=97 69.9 (60.5-79.3)	
Age, years						
	35-40	13.9 (7.3-20.6)	12.3 (1.4-23.3)	14.6 (6.3-23.0)	.78
	41-50	35.7 (25.4-45.9)	32.2 (14.2-50.3)	37.2 (24.7-49.7)	
	51-60	23.5 (16.1-30.8)	26.9 (12.4-41.4)	22.0 (13.5-30.5)	
	61-70	9.1 (3.3-14.9)	5.6 (−0.9 to 12.2)	10.5 (2.8-18.3)	
	71+	17.8 (9.4-26.2)	22.9 (5.1-40.7)	15.6 (6.6-24.7)	
Gender						
	Male	36.8 (26.6-47.0)	45.5 (26.9-64.1)	33.0 (20.7-45.4)	.15
	Female	63.2 (53.0-73.4)	54.5 (35.9-73.1)	67.0 (54.6-79.3)	
Race/ethnicity						
	Black, non-Hispanic	55.2 (46.4-63.9)	46.3 (30.3-62.3)	59.0 (49.0-69.0)	.32
	Hispanic	29.4 (19.4-39.4)	40.5 (22.6-58.4)	24.6 (12.7-36.5)	
	Other	15.4 (8.4-22.4)	13.1 (1.4-24.9)	16.4 (7.9-25.0)	
Income, US $						
	<$25K	34.2 (24.8-43.5)	32.6 (16.7-48.5)	34.9 (23.3-46.5)	.41
	$25K-$49K	33.9 (24.4-43.5)	36.4 (18.0-54.7)	32.9 (21.7-44.0)	
	$50K-$99K	17.1 (8.9-25.3)	10.3 (−1.1 to 21.7)	20.1 (9.5-30.6)	
	≥$100K	5.8 (1.2-10.4)	4.3 (−1.8 to 10.3)	6.5 (0.4-12.5)	
	Don't know/refused	9.0 (2.4-15.5)	16.5 (0.5-32.4)	5.7 (−0.4 to 11.9)	
Education						
	Middle school/ some high school	27.2 (17.8-36.6)	49.5 (30.8-68.1)	17.5 (8.0-27.1)	.01
	High school graduate/GED^d^	36.2 (26.1-46.3)	24.6 (9.0-40.1)	41.2 (28.7-53.7)	
	Associates/ some college	36.6 (27.4-45.9)	26.0 (11.1-40.8)	41.2 (29.6-52.8)	
Employment status						
	Unemployed	14.7 (8.0-21.4)	20.3 (5.3-35.3)	12.3 (5.3-19.2)	.22
	Employed	42.5 (32.3-52.6)	37.5 (20.0-55.1)	44.6 (32.2-56.9)	
	Retired	18.6 (10.6-26.6)	8.9 (0.5-17.4)	22.8 (12.1-33.5)	
	Unable to work	12.5 (5.9-19.1)	20.2 (3.2-37.2)	9.1 (3.8-14.5)	
	Other	11.8 (4.9-18.7)	13.0 (0.7-25.3)	11.2 (2.8-19.7)	
Health insurance						
	Uninsured	17.1 (9.7-24.5)	14.0 (0.8-27.3)	18.4 (9.3-27.5)	.56
	Medicaid only	13.7 (6.9-20.6)	21.5 (5.9-37.1)	10.4 (3.6-17.2)	
	Medicare only	10.8 (5.7-16.0)	9.9 (1.4-18.4)	11.2 (4.8-17.7)	
	Private/other	40.5 (30.5-50.5)	33.5 (16.9-50.0)	43.5 (31.6-55.4)	
	Multiple	17.9 (9.8-25.9)	21.1 (3.6-38.6)	16.5 (7.7-25.2)	
Physician visit in past year (% yes)		82.5 (73.6-91.4)	79.3 (63.7-95.0)	83.9 (72.9-94.8)	.63
Source of regular care (% yes)		96.7 (93.1-1.0)	89.1 (77.3-100)	100 (100-100)	.01

^a^ A total of 12 people were missing a body mass index value. These individuals were excluded from the chi-square analysis.

^b^ BMI: body mass index.

^c^*P* value for comparison between undiagnosed and diagnosed obese populations; significant at level *P*<0.05.

^d^ GED: general educational development.

### ICT Use, Measured Obesity, and Self-Reported Diagnosis of Comorbid Chronic Conditions

Among people with measured obesity, a self-reported diagnosis of hypertension (46.8%) or diabetes (16.8%) was prevalent. Only 36.8% of people with measured obesity had no diagnosis of diabetes or hypertension; 20.5% of the population had all 3 conditions. Isolated measured obesity was associated with higher rates of health-specific ICT use than measured obesity plus comorbid diabetes and/or hypertension diagnosis (77.1% obesity only vs 47.4% obesity and hypertension or diabetes vs 60.7% obesity, hypertension, and diabetes, *P*=.04; Table 4). Examining rates of specific health-related activities, a statistically significant difference was only noted for accessing Web-based health benefits information (27.6% obesity only vs 18.0% obesity and hypertension or diabetes vs 4.0% obesity, hypertension, and diabetes, *P*=.04).

**Table 4:** Comparing information and communication technology activities by presence of comorbid conditions (results of weighted analysis).

ICT^a^ activities (% yes)		Measured obesity only^c^ % (95% CI)	Measured obesity and hypertension or diabetes % (95% CI)	Measured obesity, hypertension, and diabetes % (95% CI)	*P* ^b^
		n=44 36.8 (26.6-47.0)	n=68 42.7 (32.8-52.6)	n=28 20.5 (12.4-28.6)	
General ICT use		93.7 (87.2-100)	75.0 (60.3-89.6)	73.4 (53.1-93.7)	.05
Any health-specific use		77.1 (61.4-92.7)	47.4 (33.1-61.8)	60.7 (39.3-82.0)	.04
	Seek health info online	67.5 (49.8-85.2)	45.9 (31.7-60.0)	48.4 (26.6-70.3)	.15
	Use Web-based resources to avoid asking doctor	30.8 (15.1-46.5)	21.9 (10.5-33.4)	15.6 (−1.2 to 32.4)	.41
	Web-based access of health benefit info	27.6 (12.4-42.8)	18.0 (7.1-28.9)	4.0 (−1.8 to 9.8)	.04
	Participate in online health support group	15.3 (1.7-28.9)	10.5 (1.1-19.8)	2.0 (−2.0 to 6.0)	.22
	Web-based access of health records	16.4 (4.2-28.6)	9.1 (2.0-16.1)	13.7 (−1.3 to 28.7)	.57
	Web-based medication purchasing	10.8 (0.78-20.7)	8.1 (1.4-14.7)	12.8 (−2.0 to 27.5)	.81
	Web-based communication with providers	13.1 (1.8-24.5)	10.2 (3.0-17.4)	15.7 (−1.0 to 32.4)	.80
	Use health-related mobile app	9.7 (0.8-18.6)	11.7 (2.2-21.3)	2.0 (−2.0 to 6.0)	.29

^a^ ICT: information and communication technology.

^b^*P* value for comparison between those with “measured obesity only,” “measured obesity and hypertension or diabetes,” and “measured obesity, hypertension, and diabetes”; significant at level *P*<0.05.

^c^ A total of 12 people were missing a body mass index value. These individuals were excluded from the chi-square analysis.

## Discussion

This study describes ICT use in high-poverty African American and Hispanic communities on Chicago’s South Side with a disproportionate burden of obesity and obesity-related diseases and examines the association between obesity and ICT use. To our knowledge, this is the only study to ascertain measured BMI, self-reported obesity diagnoses, and ICT use from the same sample. This design, albeit limited by a relatively small sample size, enabled us to generate three new findings. First, we found that ICT use patterns differed by measured obesity status; people with obesity had statistically significant higher rates of both general ICT and health-specific ICT use compared with people without obesity. Second, among people with measured obesity, a physician’s diagnosis of obesity was not associated with higher rates of health-specific ICT use or use of Web-based health-related information sources, but it was associated with a higher rate of using Web-based resources to avoid asking questions of a doctor. Finally, an unexpected association between comorbidity burden and health-specific ICT use was found. The highest rates of ICT use were among people with measured obesity only, as compared with those with measured obesity who reported one or two common comorbidities.

In contrast to the Pew study findings that showed lower ICT use among people with other common chronic conditions [14], our study found obesity to be associated with a higher likelihood of general ICT use. Obesity, for some, is associated with difficulties with mobility and other physical activities—factors both potentially related to increased rates of ICT use [27-30]. The physical limitations imposed by obesity may result in a vicious cycle of restricted mobility, greater use of ICT, increasing weight, and increasing physical limitation. These problems are likely exacerbated in high-poverty communities with limited access to health care and other health-promoting resources (eg, fresh food, safe spaces for exercise) [3]. The new finding of relatively high rates of health-specific ICT use among people with obesity in a high-poverty urban community may just reflect greater use of all types of ICT.

Unlike other chronic conditions (eg, hypertension and diabetes), obesity is an outward-facing condition. The social stigma of obesity may lead individuals to ICT-based resources rather than medical care for their health needs [31]. Our findings of higher use of Web-based information resources to avoid asking doctors, among those with diagnosed obesity, along with past work in other stigmatized health conditions, support this possibility. A national survey of adult Internet users found that users with a stigmatized condition (eg, depression, sexually transmitted diseases) were more likely than those with a less stigmatized condition (eg, diabetes, back pain) to report using the Internet as a health information source and as a tool to communicate with clinicians [32]. Obesity was not included among the stigmatized conditions in this study.

Regardless of the drivers behind increased health-specific ICT use, our study suggests that obesity may be a useful target for health-specific ICT-based interventions. The high prevalence of obesity among residents on Chicago’s South Side and in other high-poverty, minority communities, along with the high rate of health-specific use, indicates an already online population with high health needs and risks. ICT-based resources could potentially not only aid in the self-care and management of obesity but also serve as an entry point to provide information and support for routine preventive care and other important health topics in this population.

Among studies of health-related ICT use, this survey was unique in its combination of ICT use measures with anthropometric measures and assessment of self-reported chronic diseases [14,33]. This design enabled closer study of the association between physician-diagnosed obesity and health-specific ICT use. Counter to our hypothesis, obese people who reported a physician’s diagnosis of obesity were not more likely to report health-specific ICT use. This result differs from past evidence demonstrating the effect of physician input on health behaviors. In a randomized controlled trial of adults in a primary care setting, individuals randomized to receiving physicians’ advice on quitting smoking, reducing fat consumption, and increasing exercise were more likely to believe these topics were relevant to them and more likely to report attempting to quit smoking and making some dietary changes [34]. The absence of differences in health-specific ICT use by provider diagnosis may again reflect differences in obesity as a condition. Unlike diabetes, dyslipidemia, and hypertension diagnosed by blood test or expert measurement, obesity is easily self-diagnosed making the physician’s diagnosis less surprising and, potentially, less important.

Obesity status may also be more subject to individual perceptions than other chronic conditions. Past research has demonstrated that people have difficulty in assessing ideal weight [35]. Beyond that, differences in obesity perception by race and ethnicity are well documented in the literature. Using pooled cross-sectional data from National Health and Nutrition Examination Survey, Dorsey et al [36] observed differences in weight perception when comparing non-Hispanic black adults to non-Hispanic white adults; non-Hispanic black adults with obesity who did not perceive themselves as obese had lower odds of desiring weight loss. Given this, it may be that a physician’s obesity diagnosis has a different effect when it is discordant with existing cultural norms and an individual’s perception of his or her weight. If that is the case, the need to seek out health resources for obesity, including ICT-based health resources, may be less affected by a provider’s diagnosis.

Comorbid diagnoses of diabetes and/or hypertension were not found to be associated with a higher likelihood of health-specific ICT use among individuals with measured obesity. This finding contrasts with findings from a 2007 phone survey of US adults that demonstrated a positive correlation between the number of chronic conditions (did not include obesity) and engagement in selected health-specific ICT activities [37]. However, this study’s population was younger (>60% aged less than 50 years), had higher educational attainment, and a much lower prevalence of chronic conditions. Also, the racial and ethnic characteristics of the sample were not described. Individuals with obesity in addition to hypertension and/or diabetes are likely to have more frequent in-person contact with health care providers than individuals with obesity alone and may better understand obesity as, or relating to exacerbation of, a chronic medical condition. It is possible that among individuals with obesity and fewer comorbid conditions, less regular provider contact may result in more unanswered health questions and unmet needs, motivating more health-specific ICT use.

**Figure 2:**
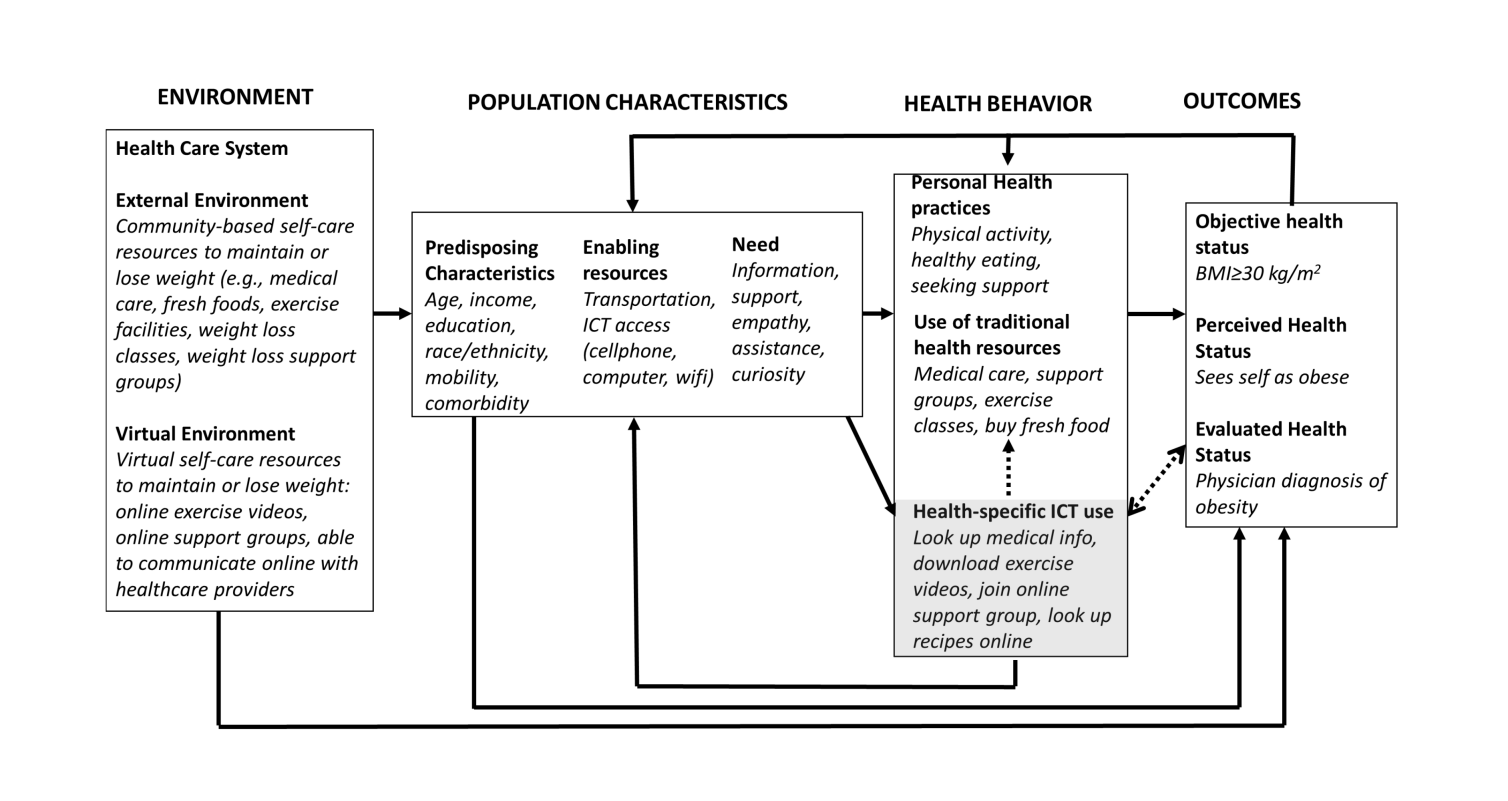
Proposed conceptual framework for the relationship between obesity and health-specific information and communication technology (ICT) use derived from literature, study results, and clinical experience. Adapted from Andersen’s Behavioral Model of Health Services Utilization, the proposed model incorporates obesity as a specific use case. The dashed lines highlight two incompletely understood domains: (1) the relationship between obesity and health-specific ICT use and (2) the potential dual role of health-specific ICT as both an access point to and a replacement for traditional health resources. BMI: body mass index.

On the basis of the study findings, extant literature, and clinical experience, we propose a preliminary conceptual framework for the relationship between obesity and use of health-specific ICT (Figure 2) [14,20,28,29,31,36,38,39]. The proposed framework is adapted from Andersen’s Model of Health Services Utilization [40]; it incorporates obesity as a specific use case and highlights the still incompletely understood interplay between obesity and ICT use. The proposed model also demonstrates the potential for ICT-based services to act as both a complement to traditional health services by enabling access (eg, an individual uses the Internet to find a weight loss support group) and a partial or complete substitute for traditional health services (eg, individual may use the Internet to connect with a weight loss support group online).

The study has several limitations. First, the conservative AAPOR response rate calculation (44.6%) is lower than desired, but it is also consistent with or higher than that reported for other similar surveys in this and other urban populations [41-43]. Of note, Pew reports an 11.6% response rate for its widely cited 2012 phone survey (although used different sampling approach, random digit dialing) [14,44]. Second, although the use of high-quality population-based probability sampling serves to balance the relatively small sample size and allow for generalization of the findings beyond just the survey respondents, the racial and ethnic characteristics of the studied population may not generalize to other groups. Next, although we were able to report on broad categories of health-specific technology use in this population, we did not collect comprehensive information on exactly how and why people were using health-related ICT. It is also possible that we did not capture all of the current health-specific ICT activities in which people may engage. While we describe an association between measured obesity and health-specific ICT use, we cannot infer causality using cross-sectional data. As has been postulated, it is possible that higher levels of any type of ICT use (including health-specific use) cause sedentariness and increase the likelihood of obesity [29,30].

In this high-poverty urban population, the majority of people with measured obesity reported use of technology for health-specific reasons. This high-risk, already online population presents an opportunity for ICT-based health resources to impact health, especially in communities where the burden of obesity is high. However, understanding current use patterns and potential opportunities for health-specific ICT-based resources is only a first step. The critical next step is evaluating the ability of these technology-based resources to meaningfully impact health care and health outcomes in this high-need, high-risk population.

## Appendix

Multimedia Appendix 1 Frequency of health-specific information and communication technology activities.

## Abbreviations

AAPOR: American Association for Public Opinion Research
BMI: body mass index
ICT: information and communication technology
IRB: Institutional Review Board
SSHVS: South Side Health and Vitality Studies
GED: general educational development
