# Supplementary material for: Health-Specific Information and Communication Technology Use and Its Relationship to Obesity in High-Poverty, Urban Communities: Analysis of a Population-Based Biosocial Survey
Source: J Med Internet Res. 2016 Jun 28;18(6):e182. doi: 10.2196/jmir.5741 (PMC4942684; doi:10.2196/jmir.5741)
Supplement: Multimedia Appendix 1 [file jmir_v18i6e182_app1.pdf]

Multimedia Appendix 1: Frequency of health-specific ICT activities (%[95%CI])

Health-specific ICT activities[a]	Every day	At least once per week	At least once per month	Less than once per month	Never
**Seek health info online**	4.8(1.7-7.8)	11.7(7.2=16.2)	14.3(9.6-19.0)	16.5(11.00-21.9)	52.8(45.5-60.0)
**Access health benefit info online**	0.5(-0.5-1.5)	0.6(-0.6-1.8)	2.4(0.5-4.2)	8.7(5.0-12.5)	87.8(83.4-92.2)
**Participant in online health support group**	0	1.1(-0.1-2.8)	1.4(0.1-2.6)	6.8(3.2-10.5)	90.7(86.6-94.9)
**Access online health records**	0	0	3.1(0.5-5.6)	5.7(2.7-8.8)	91.2(87.3-95.1)
**Purchase medications online**	0	0.6(-0.3-1.5)	2.6(0.6-4.6)	4.5(1.8-7.2)	92.3(88.9-95.7)
**Communicate with providers online**	0	0.9(-0.3-2.2)	1.1(-0.1-2.4)	7.6(4.2-11.1)	90.3(86.5-94.1)

[a]Information on use frequency was not collected for the following activities: “Use of online resource to avoid asking doctor” and “Use of health-related mobile apps”
